# Oxidant Status and Lipid Composition of Erythrocyte Membranes in Patients with Type 2 Diabetes, Chronic Liver Damage, and a Combination of Both Pathologies

**DOI:** 10.1155/2013/657387

**Published:** 2013-06-06

**Authors:** Rolando Hernández-Muñoz, Marisela Olguín-Martínez, Irma Aguilar-Delfín, Lourdes Sánchez-Sevilla, Norberto García-García, Mauricio Díaz-Muñoz

**Affiliations:** ^1^Departamento de Biología Celular, Instituto de Fisiología Celular, UNAM, 04510 México DF, Mexico; ^2^Instituto Nacional de Medicina Genómica (INMEGEN), 14610 México DF, Mexico; ^3^Departamento de Neurobiología Celular y Molecular, Instituto de Neurobiología, Campus UNAM, 76230 Juriquilla QRO, Mexico

## Abstract

There is an important set of cirrhotic and diabetic patients that present both diseases. However, information about metabolic and cellular blood markers that are altered, in conjunction or distinctively, in the 3 pathological conditions is scarce. The aim of this project was to evaluate several indicators of prooxidant reactions and the membrane composition of blood samples (serum and red blood cells (RBCs)) from patients clinically classified as diabetic (*n* = 60), cirrhotic (*n* = 70), and diabetic with liver cirrhosis (*n* = 25) as compared to samples from a similar population of healthy individuals (*n* = 60). The results showed that levels of TBARS, nitrites, cysteine, and conjugated dienes in the RBC of cirrhotic patients were significantly increased. However, the coincidence of diabetes and cirrhosis partially reduced the alterations promoted by the cirrhotic condition. The amount of total phospholipids and cholesterol was greatly enhanced in the patients with both pathologies (between 60 and 200% according to the type of phospholipid) but not in the patients with only one disease. Overall, the data indicate that the cooccurrence of diabetes and cirrhosis elicits a physiopathological equilibrium that is different from the alterations typical of each individual malady.

## 1. Introduction

Diabetes mellitus (DM) is a worldwide disease frequently associated with a high risk of atherosclerosis and renal, cerebral, and ocular damage [1]. Oxidative damage plays several roles in diabetes and its complications [1–3], and reactive oxygen species (ROS) have been implicated in the pathogenesis of DM [4]. Patients with type 2 DM frequently have vascular endothelium dysfunction associated with hypercholesterolemia. It has been reported that patients with type 2 DM, hypertension, cirrhosis, and malaria show a nitric oxide (NO) deficiency as a major factor contributing to endothelial dysfunction [5].

In the same context, increased production of ROS has been related to protein glycosylation [2] and/or glucose autooxidation in DM patients [6]. Glycosylated proteins differ in their biological half-lives and properties. Glycosylated serum albumin reflects glycemia levels, since hemoglobin undergoes increased glycosylation (Hb A_1C_) throughout the life span of the red blood cells (RBC) under hyperglycemic conditions [7]. Glycosylation of proteins can lead, in turn, to oxidative stress by direct release of superoxide and H_2_O_2_ [8]. Glycosylated albumin is a more sensitive index of short-term variations of glycemia than Hb A_1C_ during treatment of diabetic patients [9]. High plasma malondialdehyde (TBARS) and organic hydroxyperoxide concentrations have been observed in patients with ketoacidosis as secondary effects of glycemic disorders [10]. Additionally, increased lipid peroxidation (LP) occurs in RBC membranes due to an excessive production of ROS and decreased levels of GSH. Hematological alterations in plasma and/or blood cells (high serum levels of conjugated dienes and lipid peroxides) have been observed in type 2 DM patients with vascular complications [11].

It is not infrequent to find an association of DM with several modalities of liver disease. Diabetes and liver injury appear to be associated [12]: elevated levels of both alanine and aspartate aminotransferases occur in diabetics more frequently than in the general population [13], even independently of obesity [14], as well as serum *γ*-glutamyltransferase activity, which has been proposed as a marker of insulin resistance in type 2 DM [15]. Causes of cirrhosis linked to diabetes include nonalcoholic fatty liver disease, hemochromatosis, and hepatitis C infection. Taken together, these data are highly suggestive of a DM effect on liver functions [16]. 

Nonalcoholic fatty liver disease (NAFLD) represents a spectrum of progressive liver maladies encompassing simple steatosis, nonalcoholic steatohepatitis (NASH), fibrosis, and cirrhosis. NAFLD is strongly associated with glucose intolerance or type 2 DM. Importantly, accumulating evidence indicates that NAFLD is strongly associated with a prothrombotic tendency, which may, at least in part, contribute to the increased risk of atherothrombotic events observed in these patients. NAFLD also exacerbates systemic and hepatic insulin resistance and causes atherogenic dyslipidemia [17]. Also recently, it was reported that interleukin-2R, interleukin-18, and glucagon are higher in DM patients with cirrhosis, suggesting a synergistic effect of both diseases [18]. Moreover, interactions between diabetes and hepatitis C virus exacerbated the liver damage, suggesting that diabetes is a risk factor for the pathological progression of the viral liver disease [19]. 

 Indeed, it has been suggested that the combination of insulin resistance and LP could lead to liver damage, such as those in NASH [20]. Hence, this study was addressed to evaluate the impact of type 2 DM combined with advanced liver damage (diagnosed as cirrhosis) on parameters indicative of oxidative stress and its impact on biological membranes as measured in the patients' RBC.

## 2. Material and Methods

### 2.1. Patients and Controls

Subjects with different stages of type 2 DM were recruited from outpatient clinics as coordinated by the Instituto Nacional de Medicina Genómica (INMEGEN). Patients diagnosed with cirrhosis, with or without manifested clinical type 2 DM, were recruited from several outpatient clinics of the Sector Salud (Ministry of Health). The study involved 60 patients with type 2 DM, 70 patients with cirrhosis, 25 patients with both pathologies. Patients were selected based upon the following criteria: all of them were nonalcoholics, nonsmokers, and apparently free from any renal complication. The control group consisted of healthy individuals of similar age, body weight matched, nonsmoking, nonalcoholic, and with no family history of diabetes and/or cirrhosis. Following a 12 h overnight fast, all subjects were blood sampled and clinically evaluated by the same investigators (Norberto García-García and Irma Aguilar-Delfín). This study was carried out in accordance with the Declaration of Helsinki (2000) of the World Medical Association and approved by the Ethics Committees of the Hospital General de México (Ministry of Health) and Instituto de Fisiología Celular (UNAM). 

### 2.2. Clinical Tests

In separate blood samples from healthy subjects and diabetic patients, several clinical parameters were quantified: glucose, glycosylated Hb A_1C_, cholesterol, triacylglycerols, C-reactive protein (CRP), albumin, bilirubin, coagulation factors, aspartate (GOT) and alanine (GPT) aminotransferase activities, and *γ*-glutamyltransferase (GGT) activity.

### 2.3. Blood Samples

Heparin-anticoagulated blood was obtained, and the serum was rapidly separated. Aliquots of serum and RBC were placed in ice cold perchloric acid (8% w/v, final concentration). After centrifugation, acid extracts of plasma as well as of RBC were obtained (dilution: 1 : 3 v/v blood samples/perchloric acid) and stored at −50°C until use.

### 2.4. Biochemical Measurements

In acid extracts from whole blood, serum, and RBC, thiobarbituric acid reactive substances (TBARS) were determined by the method described by Hernández-Muñoz et al. [21], and free cysteine was colorimetrically assayed with the method described by Gaitonde [22]; in neutralized perchloric acid extracts, nitrites were quantified by the Griess reaction [23]. Cell membrane LP related and conjugated dienes (CD) were assessed as previously described [24], and the protein carbonyl content in the different subcellular fractions was estimated according to Levine et al. [25] as an index of oxidative damage. Total hemoglobin was quantified using Drabkin's reagent.

### 2.5. Preparation of RBC Membranes (Ghosts)

Sets of anticoagulated blood samples were obtained, and the serum was rapidly separated by centrifugation at 900 g for 5 min at 4°C. The buffy coat was removed, and the erythrocyte pellet was washed 4 times with 2 volumes of cold (4°C) 20 mM HEPES (pH 7.4) containing 0.9% NaCl. Thereafter, RBC were gently resuspended in a hypoosmotic solution containing 0.172 M TRIS buffer (pH 7.6) and adjusted to a 50% hematocrit to produce hemolysis. RBCs were then centrifuged at 20,000 g (4°C) for 25 min then washed at least 3 more times to completely remove hemoglobin from the RBC pellet, as described before [26]. Thereafter, membranes were incubated in the same HEPES buffer for 30 min at 37°C, and TBARS was measured [21].

### 2.6. Calculations and Statistics

Concentration of serum and RBC metabolites were calculated as nmoles/mL and expressed as means ± standard deviation (SD). To compare a continuous variable between groups, the Student's unpaired *t*-test and the Mann-Whitney test were used; thereafter, these differences were contrasted with a *t*-test for paired data. 

## 3. Results

### 3.1. Metabolites Indicating Oxidative Stress and Generation of NO in Patients with Type 2 DM and Cirrhosis

**Figure 1:**
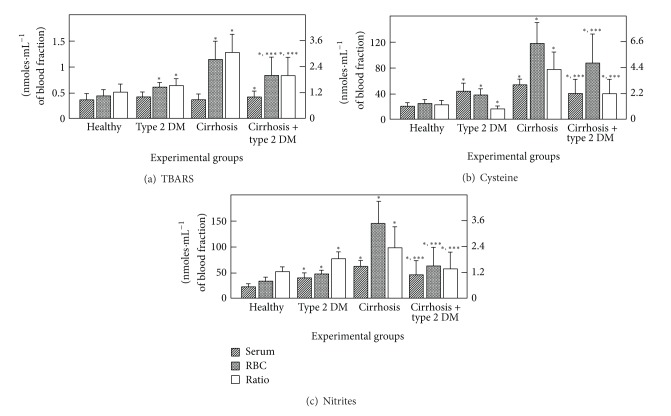
Blood levels of TBARS, cysteine, and of nitrites and its distribution in serum and RBC from patients with type 2 diabetes mellitus and cirrhosis. The results are expressed as the mean ± SD for levels of TBARS (a), free cysteine (b), and for nitrites (c), in serum and RBC samples from control healthy volunteers (*n* = 60), patients with type 2 diabetes mellitus (*n* = 60), patients with cirrhosis (*n* = 70), and those from patients having the combination of both pathologies (*n* = 25). Symbols indicate each blood compartment at the top of the panels. RBC/serum ratio is indicated by the empty bars and assessed by the right scale. Statistical significance: *P < 0.01, versus control; **P < 0.01, against DM or versus cirrhosis; ***P < 0.01, against both, the diabetes and cirrhosis groups, separately.

RBC metabolite concentrations clearly differed from those found in serum (Table 1), suggesting that RBCs could act as a biochemical reservoir. In serum from patients with DM, the level of TBARS was not significantly different from that of the control group; however, the RBC content of TBARS was increased in samples obtained from patients with type 2 DM. The ratio RBC-TBARS/serum TBARS in controls was 1.2, whereas in DM patients it increased significantly ~25% (Figure 1(a)). In the group of cirrhotic patients, RBC-TBARS was drastically increased, while the level of TBARS in serum was practically unchanged, which led to a 3-fold increase in the RBC-TBARS/serum-TBARS ratio (Figure 1(a)). Interestingly, the combination of DM and cirrhosis enhanced serum-TBARS levels, when compared with controls. However, the TBARS level in the RBC was similar to that of the DM patients: the significant increase detected in the cirrhotic patients was not observed (Figure 1(a)).

The levels of free cysteine, which reflect glutathione synthesis and oxidative status, were found to be slightly higher in control RBC than in serum (Figure 1(b)). DM promoted an increased cysteine concentration in both serum and RBC; however, the RBC/serum ratio for this amino acid was significantly decreased, due a larger increase in serum cysteine (Figure 1(b)). On the other hand, cirrhosis induced the opposite pattern, a drastic increase of cysteine, mainly in the RBC. Again, the cooccurrence of both pathologies partially counteracted the alterations observed in the cirrhotic patients (Figure 1(b)). 

The concentration of blood nitrites, as a reflection of NO catabolism, was higher in the RBC than in serum in controls (Figure 1(c)). Patients with type 2 DM clearly showed an increased amount of blood nitrites, particularly in RBC, leading to a significantly higher RBC-nitrite/serum-nitrite ratio. Cirrhosis also promoted NO catabolism mainly in RBC, producing an even more elevated RBC-NO/serum-NO ratio (Figure 1(c)). Interestingly, the combination of the two diseases attenuated their individual effects on nitrites in both blood compartments (Figure 1(c)).

### 3.2. Oxidative Parameters and TBARS Production by Isolated RBC Membranes from Patients with Type 2 DM and Cirrhosis

**Figure 2:**
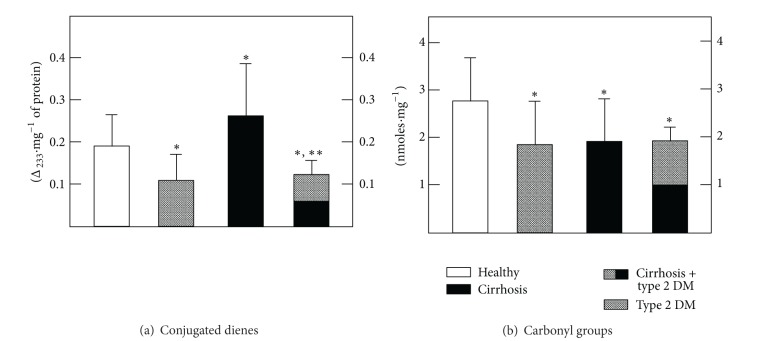
Levels of conjugated dienes and of carbonyl groups (oxidized proteins) in RBC membranes obtained from patients with type 2 diabetes mellitus and cirrhosis. The results are expressed as the mean ± SD for levels of conjugated dienes (a) and for nitrites (b), determined in isolated membranes from RBC obtained from control healthy volunteers (*n* = 60), patients with type 2 diabetes mellitus (*n* = 60), patients with cirrhosis (*n* = 70), and those from patients having the combination of both pathologies (*n* = 25). Symbols indicate experimental groups at the top of the figure. Statistics as indicated in Figure 1.

The LP rate was evaluated in RBC membranes by measuring conjugated dienes (Figure 2(a), Table 4). Compared to the TBARS generated in whole blood, RBC membranes from patients with DM had a reduced content of conjugated dienes, while those obtained from cirrhotic patients showed a significant increase of these LP by-products (Figure 2(a), Table 4). It was noteworthy that DM completely blocked the cirrhosis-induced enhancement of conjugated dienes in the RBC membranes (Figure 2(a), Table 4). The impact of oxidative stress on proteins, as assessed by the presence of carbonyl groups present in denatured membrane proteins, also changed (Figure 2(b), Table 4). When compared with controls, the RBC ghosts from patients with type 2 DM as well as RBC from cirrhotic patients had significantly lower amounts than that of carbonyl groups (Figure 2(b), Table 4). In patients with both pathologies, no additional effect was found (Figure 2(b), Table 4).

**Figure 3:**
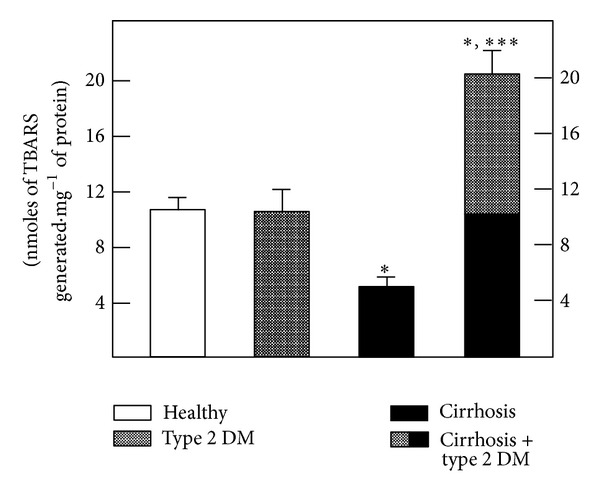
*In vitro* production of TBARS by incubated RBC membranes obtained from patients with type 2 diabetes mellitus and cirrhosis. The results are expressed as the mean ± SD for the amount of TBARS generated by isolated RBC membranes obtained from control healthy volunteers (*n* = 60), patients with type 2 diabetes mellitus (*n* = 60), patients with cirrhosis (*n* = 70), and those from patients having the combination of both pathologies (*n* = 25). Symbols indicate experimental groups at the top of the figure. Statistics as indicated in Figure 1.

 Since the cooccurrence of type 2 DM in cirrhotic patients seemed to provide some kind of antioxidant effect in the RBC, we assayed *in vitro* for TBARS generation in RBC membranes (Figure 3). TBARS synthesis in samples obtained from the cirrhotic patients was reduced, whereas the samples from DB patients did not show significant differences from the control (Figure 3). Moreover, an unexpected potentiation of TBARS production in RBC membranes was detected in samples from patients with both type 2 DM and cirrhosis (Figure 3). 

### 3.3. Changes in Lipid Composition (Phospholipids and Cholesterol) in Isolated RBC Membranes from Patients with Type 2 DM and Cirrhosis

We tested if the changes observed in the oxidative stress parameters could be correlated with alterations in the phospholipid and cholesterol content of the RBC membranes (Tables 2 and 3). The lipid composition of RBC membranes from patients with type 2 DM was practically unmodified when compared to controls (Table 2). In contrast, patients with cirrhosis had a decreased amount of phosphatidylserine (PS), which was accompanied by an elevated concentration of phosphatidylcholine (PC) and cholesterol (Table 2). Again, an unexpected pattern was found in RBC membranes from diabetic patients with cirrhosis, where all phospholipids tested and cholesterol were drastically increased (Table 2).

 Two parameters indicative of membrane fluidity were also calculated, namely, the ratio of PC/phosphatidylethanolamine (PE), as well as the total phospholipid/cholesterol ratio. Whereas in patients with DM, the PC/PE ratio was significantly decreased; in the cirrhotic patients, an increase of the membrane cholesterol level was noted, resulting in a significantly higher total phospholipid/cholesterol ratio (Table 3). These effects were completely absent in the DM and cirrhotic patients, since cirrhosis seemed to correct the DM-induced decrease of the PC/PE ratio and type 2 DM normalized the total phospholipid/cholesterol ratio in RBC membranes from cirrhotic patients (Table 3).

## 4. Discussion

Diabetes is frequently diagnosed in patients with cirrhosis and represents an important risk factor for morbidity and mortality, since pharmacological therapy is limited by hepatotoxicity and the risk of hypoglycemia. Conversely, cirrhosis is a common complication in diabetic patients. Diabetes increases the risk of fatty liver, which can progress to cirrhosis. The interactions of these pathologies are not well understood, but a possible participation of ROS as an underlying mechanism is under robust investigation. In this context, increased adiposity and insulin resistance in obese subjects contribute to the progression of NASH to fibrosis, apparently by augmenting ROS formation and altering adipokine/cytokine production, thereby promoting a profibrotic milieu in the liver [27]. 

Production of ROS promotes activation of hepatic stellate cells (HSC) and progression to fibrosis. Indeed, in a diabetic state, ROS production is enhanced in association of CYP2E1 induction and activity [28]; the resultant oxidative stress then can directly increase collagen production by activated HSC [29]. This situation confirms that oxidative stress coincides with many pathological conditions and diseases such as chronic obstructive pulmonary disease, cancer, diabetes, ischemia/perfusion, neurological disorders, atherosclerosis, hypertension, idiopathic pulmonary fibrosis, asthma, and liver diseases [30]. Although the negative impact of diabetes on the retinal, renal, nervous, and cardiovascular systems is well recognized [31], little is known about its effect on the liver. Nonetheless, it has recently been reported that hepatic deregulation in the setting of obesity is marked by oxidative stress and steatosis related to insulin resistance [29]. 

Products of lipid peroxidation, such as TBARS and other unsaturated aldehydes, can inactivate many cellular proteins, such as membrane-bound receptors and enzymes, by forming protein cross-linkages [32] which could alter cell permeability [33]. ROS can also alter the electrical charge and cross-linking of proteins, and by oxidizing specific amino acids such as cysteine and methionine, they increase susceptibility to proteolysis [34]. Moreover, free cysteine is generally the limiting amino acid for the synthesis of reduced glutathione (GSH) [35]. Thus, factors (e.g., insulin and growth factors) that stimulate cysteine (cystine) uptake by cells generally increase intracellular GSH concentrations [36]. In addition, increasing the supply of cysteine or its precursors (e.g., cystine, *N*-acetylcysteine, and L-2-oxothiazolidine-4-carboxylate) prevents GSH deficiency in humans and animals under various nutritional and pathological conditions [37].

The present data indicate that generation of TBARS was higher in RBC from patients with type 2 DM and, to a much larger extent, in the RBC from cirrhotic patients. However, in patients with both pathologies, the enhancement of TBARS was partly counteracted, probably due to the antioxidant status of these cells, since we found only a slight but significant increase in serum TBARS in samples obtained from the diabetic and cirrhotic groups (Figure 1(a)). This condition correlated well with the assessment of membrane conjugated dienes, where it was clear that cirrhosis promoted oxidative stress, which was blunted by the presence of DM (Figure 2(a)). However, the *in vitro* generation of TBARS was significantly diminished in RBC membranes obtained from patients with cirrhosis and largely counteracted by the simultaneous occurrence of DM (Table 4). The latter, which could appear somehow contradictory, might be explained by the antioxidant defense of each population of blood cells. In fact, RBC membranes from patients with type 2 DM and those obtained from cirrhotic patients both showed, separately, an important decrease of oxidized membrane proteins (carbonyl groups); this decrease was not additive when cells obtained from the group with both diseases were assayed (Figure 2(b)).

DM and cirrhosis both induced an enhanced amount of blood cysteine, which was more evident in the RBC, particularly in the patients with cirrhosis. This elevated blood cysteine was also partly attenuated by the combination of both pathologies (Figure 1(b)). It has been demonstrated that acetaldehyde, as a main product of ethanol oxidation, is bound to RBC, possibly due to thiazolidine formation with cysteine, and that the cysteine level was doubled in blood cells from alcoholic patients without severe liver damage [26]. In addition, cirrhotic patients display lower levels of plasma GSH and cysteine; on the contrary, RBC cysteine was found to increase significantly in all cirrhotic patients, particularly in alcoholics [38]. 

There is evidence that DM patients had altered NO metabolism [39], and in a rat model of cirrhosis that over-expressed caveolin-1, the interaction with eNOS and both the basal and stimulated production of NO are depressed [40]. This interaction may increase portal pressure and contribute to the malady, as occurs in cholestatic disease models where the upregulation of sinusoidal caveolin-1 and a decrease in eNOS activity were seen [41]. Our data agree with this altered NO metabolism, as evaluated by the presence of nitrites; they showed elevated nitrites in serum and RBC in diabetic and cirrhotic patients, a situation that was also partly counteracted when both pathologies occurred together (Figure 1(c)). However, we did not assess the impact of this altered NO production (i.e., production of peroxynitrites or nitrotyrosines) which could give us more insight into the possible mechanism underlying the opposing effects of the two pathologies, when they occur in the same patient.

The lipid composition (phospholipids and cholesterol) of RBC membranes obtained from the experimental groups showed some effects that can be attributed to the level of oxidative stress. PS synthesis and its translocation are ATP-dependent processes [42, 43], while the ratios PC/PE and cholesterol/total phospholipids have been related to the fluidity of a variety of membranes [44, 45]. Indeed, the ratio PS/PE (1.74 in controls; Table 2) was decreased by both pathologies. However, patients with both pathologies exhibited normal PC/PE (decreased by DM) and cholesterol/total phospholipid (increased by cirrhosis) ratios, as shown in Table 2. Both type 2 DM and cirrhosis are complex pathologies involving metabolic disturbances and adaptations, many of which are still unknown. Our data suggest that cooccurrence of both diseases instead of potentiating the severity of metabolic dysfunction somehow allows the achievement of a new metabolic status, whose significance remains to be elucidated.

Recent data support the fact of a complex interplay between the metabolic condition associated with DM and the pathologically defined as nonalcoholic fatty liver disease (NAFLD). NAFLD predicts the development of type 2 diabetes and vice versa, and each condition may serve as a progression factor for the other [46]. Hepatobiliary disease and associated mortality are increased in type 2 diabetes, and factors including fatty infiltration, microangiopathy, and direct glucotoxicity are likely to contribute to these outcomes [47]. The prevalence of type 2 DM is higher in patients with hepatic deregulation, such as NAFLD, chronic viral hepatitis, hemochromatosis, alcoholic liver disease, and cirrhosis. The development of DM in patients with cirrhosis is well recognized, and it is suggested that DM plays a role in the initiation and progression of liver injury [48]. Patients with chronic hepatitis C virus (HCV) infection have a significantly increased prevalence of type 2 DM compared to controls or hepatitis B virus-infected patients, independent of the presence of cirrhosis [49]. In addition, the levels of Hb A_1C_ and of HOMA-R are increased in DM patients with chronic liver damage and who are undergoing angiopathy [50]. Moreover, since the diabetic condition is associated with a significant increase of mortality in patients with compensated liver cirrhosis [51], there exists a consensus that cirrhosis will negatively impact DM installation and, in turn, DM could shorten the life of patients with the combined pathology. However, the incidence of diabetic retinopathy and cerebrovascular disease was significantly lower in the a diabetic/cirrhotic group compared to the type 2 DM group, probably due to the lower levels of serum lipoprotein A found in the combined group [52]. It has been also postulated a link between the development of fatty liver in which the inflammatory responses lead to the onset of diabetes type 2. In turn, the proinflammatory milieu favors that the diabetic state which in turn becomes a major contributor to progressive liver diseases such as fibrosis and cirrhosis [53]. Taken together, these results suggest that the cooccurrence of the two pathologies elicits a different physiopathological equilibrium between prooxidant reactions and antioxidant activities.

## 5. Conclusions

 Diabetes and cirrhosis are pathological conditions that become interconnected in an important number of patients. Our findings indicate that the oxidative response observed in blood markers in cirrhotic/diabetic patients is ameliorated in some parameters in comparison to the enhanced prooxidant activity promoted by cirrhosis. Another distinctive result was the increased amount of phospholipids content in the red blood cells of the patients with both illnesses. It remains to be elucidated the potential association between the oxidative response in each pathology with structural changes in blood cells.

## Figures and Tables

**Table 1:** TBARS, nitrites, and cysteine levels from serum and red blood cells obtained from control subjects and from patients with type 2 DM and/or cirrhosis.

Parameter	Healthy (*n* = 60)	Diabetes (*n* = 60)	Cirrhosis (*n* = 70)	Diab + Cirrhos (*n* = 25)
Serum TBARS	0.35 ± 0.13	0.40 ± 0.10	0.37 ± 0.09	0.42 ± 0.18
RBC TBARS	0.42 ± 11	0.62 ± 0.08*	1.15 ± 0.35*	0.84 ± 0.35^∗,∗∗∗^
RBC/serum ratio	1.20 ± 0.34	1.55 ± 0.29*	3.11 ± 0.89*	2.00 ± 0.78*

Serum nitrites	26 ± 3	37 ± 8	63 ± 25*	46 ± 18*
RBC nitrites	32 ± 6	68 ± 7*	150 ± 47*	62 ± 24*
RBC/serum ratio	1.2 ± 0.2	1.8 ± 0.3*	2.3 ± 0.9*	1.4 ± 0.4

Serum cysteine	18 ± 4	44 ± 12*	28 ± 9*	39 ± 15*
RBC cysteine	23 ± 7	37 ± 10	153 ± 52*	87 ± 34^∗,∗∗∗^
RBC/serum ratio	1.3 ± 0.3	0.8 ± 0.2	5.3 ± 1.8*	2.2 ± 0.9^∗,∗∗∗^

Results are expressed as mean ± SD of determinations done in blood samples membranes from controls (*n* = 60), patients with type 2 DM (*n* = 60), cirrhotic patients (*n* = 70), and diabetic patients with cirrhosis (*n* = 25). RBC: red blood cells. Statistical significance: *P < 0.01, versus control; **P < 0.01, versus DM or versus cirrhosis; ***P < 0.01, versus both the diabetes and cirrhosis groups, separately.

**Table 2:** Levels of phospholipids and cholesterol in RBC membranes obtained from control subjects and from patients with type 2 DM and/or cirrhosis.

Parameter	Healthy (*n* = 60)	Diabetes (*n* = 60)	Cirrhosis (*n* = 70)	Diab + Cirrhos (*n* = 25)
PS (nmoles/mg)	40 ± 14	34 ± 10	30 ± 8*	63 ± 14^∗,∗∗∗^
PI (nmoles/mg)	46 ± 10	45 ± 11	50 ± 12	126 ± 26^∗,∗∗∗^
PC (nmoles/mg)	62 ± 14	63 ± 16	77 ± 17*	194 ± 36^∗,∗∗∗^
PE (nmoles/mg)	23 ± 6	27 ± 6	26 ± 6	70 ± 10^∗,∗∗∗^
Cholesterol (nmoles/mg)	17 ± 4	18 ± 5	25 ± 6*	41 ± 6^∗,∗∗∗^

Results are expressed as mean ± SD of determinations done in RBC membranes from controls (*n* = 60), patients with type 2 DM (*n* = 60), cirrhotic patients (*n* = 70), and diabetic patients with cirrhosis (*n* = 25). PC: phosphatidylcholine; PE: phosphatidylethanolamine; PI: phosphatidylinositol; PS: phosphatidylserine; Diab + Cirrhos: diabetes + cirrhosis. Statistical significance: *P < 0.01, versus control; **P < 0.01, versus DM or versus cirrhosis; ***P < 0.01, versus both the diabetes and cirrhosis groups, separately.

**Table 3:** Relations of phospholipids and cholesterol in RBC membranes obtained from control subjects and in patients with type 2 DM and cirrhosis.

Parameter	Healthy (*n* = 60)	Diabetes (*n* = 60)	Cirrhosis (*n* = 70)	Diab + Cirrhos (*n* = 25)
Total phospholipids (nmoles/mg)	171 ± 44	168 ± 42	185 ± 44	453 ± 87^∗,∗∗∗^
PC/PE ratio	2.72 ± 0.62	2.34 ± 0.54*	2.71 ± 0.58	2.76 ± 0.46**
Cholesterol/T. phospholipids	0.10 ± 0.02	0.11 ± 0.03	0.13 ± 0.03*	0.09 ± 0.02***

Results are expressed as mean ± SD of determinations done in RBC membranes from controls (*n* = 60), patients with type 2 DM (*n* = 60), cirrhotic patients (*n* = 70), and diabetic patients with cirrhosis (*n* = 25). PC: phosphatidylcholine; PE: phosphatidylethanolamine; T: total; Diab + Cirrhos: diabetes + cirrhosis. Statistical significance: *P < 0.01, versus control; **P < 0.01, versus DM or versus cirrhosis; ***P < 0.01, versus both the diabetes and cirrhosis groups, separately.

**Table 4:** Conjugated dienes, carbonyls and TBARS generation in red blood cell membranes obtained from control subjects and in patients with type 2 DM and cirrhosis.

Parameter	Healthy (*n* = 60)	Diabetes (*n* = 60)	Cirrhosis (*n* = 70)	Diab + Cirrhos (*n* = 25)
Conjugated dienes (Δ_233_/mg)	0.19 ± 0.07	0.11 ± 0.06	0.26 ± 0.12*	0.12 ± 0.03***
Carbonyl groups (nmoles/mg)	2.73 ± 0.90	1.83 ± 0.85*	1.85 ± 0.89*	1.86 ± 0.38*
TBARS generation by RBC membranes	10.9 ± 0.9	10.5 ± 1.4	4.8 ± 1.0*	20.7 ± 1.8^∗,∗∗∗^

Results are expressed as mean ± SD of determinations done in RBC membranes from controls (*n* = 60), patients with type 2 DM (*n* = 60), cirrhotic patients (*n* = 70), and diabetic patients with cirrhosis (*n* = 25). Statistical significance: *P < 0.01, versus control; **P < 0.01, versus DM or versus cirrhosis; ***P < 0.01, versus both the diabetes and cirrhosis groups, separately.
